# TMT‐Based Quantitative Proteomic Profiling of Human Esophageal Cancer Cells Reveals the Potential Mechanism and Potential Therapeutic Targets Associated With Radioresistance

**DOI:** 10.1002/prca.202400010

**Published:** 2024-10-07

**Authors:** Aidi Gao, Chao He, Hengrui Chen, Qianlin Liu, Yin Chen, Jianying Sun, Chuanfeng Wu, Ya Pan, Sonia Rocha, Mu Wang, Jundong Zhou

**Affiliations:** ^1^ Suzhou Cancer Center Core Laboratory The Affiliated Suzhou Hospital of Nanjing Medical University Suzhou Jiangsu P.R. China; ^2^ Wisdom Lake Academy of Pharmacy Xi'an Jiaotong‐Liverpool University Suzhou Jiangsu P.R. China; ^3^ Department of Biological Sciences Xi'an Jiaotong‐Liverpool University Suzhou Jiangsu P.R. China; ^4^ Department of Molecular Physiology and Cell Signalling Institute of Systems Molecular and Integrative Biology University of Liverpool Liverpool UK

**Keywords:** esophageal squamous cell carcinoma, mass spectrometry, quantitative proteomics, radioresistance

## Abstract

**Purpose:**

The recurrence of esophageal squamous cell carcinoma (ESCC) in radiation therapy treatment presents a complex challenge due to its resistance to radiation. However, the mechanism underlying the development of radioresistance in ESCC remains unclear. In this study, we aim to uncover the mechanisms underlying radioresistance in ESCC cells and identify potential targets for radiosensitization.

**Methods:**

We established two radio‐resistant cell lines, TE‐1R and KYSE‐150R, from the parental ESCC cell lines TE‐1 and KYSE‐150 through fractionated irradiation. A TMT‐based quantitative proteomic profiling approach was applied to identify changes in protein expression patterns. Cell Counting Kit‐8, colony formation, γH2AX foci immunofluorescence and comet assays were utilized to validate our findings. The downstream effectors of the DNA repair pathway were confirmed using an HR/NHEJ reporter assay and Western blot analysis. Furthermore, we evaluated the expression of potential targets in ESCC tissues through immunohistochemistry combined with mass spectrometry.

**Results:**

Over 2,000 proteins were quantitatively identified in the ESCC cell lysates. A comparison with radio‐sensitive cells revealed 61 up‐regulated and 14 down‐regulated proteins in the radio‐resistant cells. Additionally, radiation treatment induced 24 up‐regulated and 12 down‐regulated proteins in the radio‐sensitive ESCC cells. Among the differentially expressed proteins, S100 calcium binding protein A6 (S100A6), glutamine gamma‐glutamyltransferase 2 (TGM2), glycogen phosphorylase, brain form (PYGB), and Thymosin Beta 10 (TMSB10) were selected for further validation studies as they were found to be over‐expressed in the accumulated radio‐resistant ESCC cells and radio‐resistant cells. Importantly, high S100A6 expression showed a positive correlation with cancer recurrence in ESCC patients. Our results suggest that several key proteins, including S100A6, TGM2, and PYGB, play a role in the development of radioresistance in ESCC.

**Conclusions:**

Our results revealed that several proteins including Protein S100‐A6 (S100A6), Protein‐glutamine gamma‐glutamyltransferase 2 (TGM2), Glycogen phosphorylase, brain form (PYGB) were involved in radio‐resistance development. These proteins could potentially serve as biomarkers for ESCC radio‐resistance and as therapeutic targets to treat radio‐resistant ESCC cells.

**Abbreviations**
CCK8: Cell Counting Kit‐8
DDR: DNA damage repair
DSB: DNA double‐strand break
ESCC: esophageal squamous cell carcinoma
FC: fold change
FDR: false discovery rate
HR: homologous recombination
IHC: immunohistochemistry
IPA: Ingenuity Pathway Analysis
NHEJ: non‐homologous end joining
PCA: principal component analysis
PE: planting efficiency
PYGB: glycogen phosphorylase, brain form
RT: radiotherapy
S100A6: S100 calcium‐binding protein A6
SER: sensitive enhancement ratio
SF: survival fraction
siNC: noncoding small interfering RNA negative control
TGM2: glutamine gamma‐glutamyltransferase 2
TMSB10: thymosin beta‐10
TMT: tandem mass tagging

## 1 Introduction

The incidence and mortality rate of esophageal squamous cell carcinoma (ESCC) in China exceeds more than half of the global average [1, 2]. The 5‐year survival rate is only 20%–30% [2, 3, 4]. Despite the advancement of radiation therapy for ESCC [2, 5], a lot of ESCC patients still face relapse or recurrence [5, 6]. Radioresistance is considered a prominent factor contributing to ESCC's poor prognosis [4, 6]. At present, the exact mechanism underlying the development of radiotherapy (RT) resistance in esophageal cancer cells remains unclear.

Presently, one of the main treatment options for ESCC include, for resectable esophageal cancer, neoadjuvant chemoradiotherapy followed by surgery is the standard treatment; for unresectable esophageal cancer, definitive chemoradiotherapy is the only curative option. Radiation therapy is typically administered once daily for several weeks, with each session delivering an approximate dosage of 2 Gy. Following five consecutive days of radiation, there is a scheduled 2‐day break, and this constitutes one treatment cycle. The total radiation dose patients received is around 60 Gy [5, 7]. The establishment of radiation‐resistant cell models after receiving the entire radiation therapy may offer insights into the development of radioresistance. Following this treatment protocol, we established a panel of radioresistant ESCC cell lines that survived following exposure to a cumulative dose of 60 Gy [7, 8, 9]. These cell lines were then subjected to a daily dose of 2 Gy x‐ray radiation for five consecutive days, resulting in acumulative dose of 10 Gy, to investigate the differential protein expression patterns using quantitative mass spectrometry.

The advancement of proteomics technology has enabled us to identify widespread changes in protein expression levels in tumor cells or tissues and uncover the protein variations associated with tumor initiation and progression [10, 11, 12, 13]. Quantitative mass spectrometry has been increasingly utilized in investigating the mechanisms of RT resistance in ESCC, as well as in the identification of potential biomarkers and therapeutic targets using stable isotope labeling in cell culture (SILAC) and isobaric labeling strategies like tandem mass tagging (TMT) [14, 15, 16]. Recent studies utilizing proteomics have revealed the molecular mechanisms and potential therapeutic targets of tumor resistance and radiation therapy resistance. Panizza et al. used liquid chromatography‐tandem mass spectroscopy (LC‐MS/MS) proteomic analysis to uncover a mechanism mediating radiation therapy resistance in gliomas, and modulation of proteomic levels can predict the radiation resistance of glioma stem cell lines GSCs [17]. Label‐free proteomics indicated that activation of the signaling transduction pathways may serve as potential mechanisms for chemoradiotherapy resistance in small cell lung cancer [18]. Furthermore, research based on TMT proteomics analysis revealed potential biomarkers that synergistically promote radiation resistance in glioblastoma [19]. These findings provide valuable insights into the tolerance of tumors to radiation therapy and present the potential for developing treatment strategies for tumor cell radiation tolerance. Among the array of techniques available, the TMT‐labeled proteomic approach has emerged as one of the most widely used and preferred options for unbiased global protein profiling in various cancer types, such as colorectal cancer, gastric cancer, and ESCC [14, 20, 21]. This highly sensitive and accurate method demonstrates exceptional efficiency in labeling and provides comprehensive coverage of the proteomes, making it particularly suitable for initial investigations into the discovery of potential biomarkers.

In this study, we employed a TMT‐based quantitative proteomics approach to differentiate between ESCC cells that are sensitive or resistant to RT, with the aim of identifying potential biomarkers or therapeutic targets for RT resistance in ESCC. Our ultimate goal is to identify a panel of protein biomarker candidates for the purpose of diagnosis, prognosis, and potentially the advancement of innovative treatments for radiation resistance in ESCC.

## 2 Materials and Methods

### 2.1 Cell Lines and Clinical Specimens

The human ESCC cell lines TE‐1 and Kyse‐150 were obtained from the Cell Bank of the Chinese Academy (Shanghai, China) and ATCC (Manassas, VA, USA). TE‐1 cells were cultured in DMEM medium, while Kyse‐150 cells were cultured in RPMI‐1640 medium. All the media used (Gibco, 6123176 and 6123046) were supplemented with 10% fetal bovine serum (FBS, Biological Industries, 04‐001‐1ACS). The cells were cultured in a humidified environment with 5% CO_2_ at a temperature of 37°C.

Paraffin‐embedded tissue samples from 60 ESCC patients in the study were obtained from Nanjing Medical University Affiliated Suzhou Hospital from 2010 to 2017 (Suzhou, Jiangsu, China). The ESCC patients were all received RT followed by surgery. All tissue specimens were promptly snap‐frozen upon acquisition and preserved at −80°C to ensure optimal sample quality. The study was approved by the Institutional Ethics Committee of the Nanjing Medical University. Patients and sample collection were all in accordance with ethical guidelines.

### 2.2 Irradiation of Cells

A 6 MV x‐ray beam was generated by a clinical linear x‐ray accelerator (Varian, TrueBeam, USA) in the Department of Radiation Oncology at Nanjing Medical University Affiliated Suzhou Hospital (Suzhou, Jiangsu, China). The parameters were set as follows, the dose rate was 200 cGy/min, the treatment field was 40 × 40 cm^2^, and the source‐to‐sample distance was 100 cm. A 1.5 cm‐thick bolus material was placed on the cover during irradiation.

**Summary**

- This study revealed that TMT‐labeled quantitative proteomics technology can distinguish differences in protein expression profiles between radio‐sensitive and radio‐resistant cells in esophageal squamous cell carcinoma (ESCC) after various radiotherapy regimens. By using two models of radio‐resistant ESCC cells, it was observed that certain proteins, such as S100 calcium‐binding protein A6 (S100A6), transglutaminase 2 (TGM2), and brain‐type glycogen phosphorylase (PYGB), have higher expression levels in resistant cells compared to sensitive cells. These proteins are involved in DNA double‐strand break repair pathways, including non‐homologous end joining (NHEJ) and homologous recombination (HR). The suppression of these proteins' expression can increase the sensitivity of ESCC cells to radiotherapy by reducing their DNA repair capacity, thereby enhancing cellular radiosensitivity. The results showed the differential protein expression profiles between radio‐sensitive and radio‐resistant cells and revealed mechanisms underlying cellular responses to radiotherapy, suggesting that modulating specific targets and associated signaling pathways may improve therapeutic outcomes in ESCC.

### 2.3 Radioresistant Cell Line Generation

Radioresistant cell lines TE‐1R and Kyse‐150R were derived from their respective parental cell lines, TE‐1 and Kyse‐150. The establishment of the TE‐1R cell line has been previously described [22]. For this study, the Kyse‐150 cells were subjected to three treatment cycles, each consisting of exposure to a daily dose of 2 Gy x‐ray radiation for five consecutive days, followed by a 2‐day break. The cumulative dose reached 60 Gy, which is comparable to the dosage used in the clinical management of ESCC patients. With the increase in radiation dosage, a large portion of ESCC cells underwent apoptosis, while a certain number of cells persisted and underwent proliferation. Harvest the cells at different ionizing radiation (IR) dosages for protein extraction during the cell passage process.

### 2.4 Clonogenic Survival Assay

Clonogenic survival assay was performed as described previously [22, 23]. Cells were seeded following an extreme limiting dilution (200, 1000, 2000, 6000, and 12,000 per well) into 6‐well plates. Subsequently, they were irradiated with x‐rays at doses of 0, 2, 4, 6, and 8 Gy, respectively. After being cultured for about 10−14 days, plates were stained with Giema Wright solution. Then, the number of colonies was calculated and analyzed by image J software (v1.52a, National Institutes of Health, NIH, Bethesda, MD, USA). The determined value and surviving fraction were obtained according to the multitarget single‐hit mathematical model SF = 1−(1 − exp(−*D*/*D*_0_)*N*. Sensitive enhancement ratio (SER) = $\left(D_{0}\;\mathrm{of}\;\mathrm{the}\;\mathrm{control}\;\mathrm{group}\right)/\left(D_{0}\;\mathrm{of}\;\text{the}\;\mathrm{experimental}\;\mathrm{group}\right)$. *D*: the dose of radiation. *D*_0_: *D*: the dose of radiation. *D*_0_: the average lethal dose, which is the radiation amount needed to kill 63% of cells on the survival curve. *D*_q_: the width of the curve at the point of intersection between the extrapolated linear section of the survival curve (under the non‐single‐hit model) and the line of survival fraction equal to 1 (the shoulder width). The curve was fitted using Graph Prism 8 software (Dotmatics, Boston, MA, USA).

The planting efficiency (PE) is calculated as follows:

$$\mathrm{PE}=\frac{\;\mathrm{number}\;\mathrm{of}\;\mathrm{colonies}\;\mathrm{formed}\;\mathrm{in}\;\mathrm{the}\;0\;\mathrm{Gy}\;\mathrm{group}}{\;\mathrm{number}\;\mathrm{of}\;\mathrm{cells}\;\mathrm{seeded}\;\mathrm{in}\;\mathrm{the}\;0\;\mathrm{Gy}\;\mathrm{group}}$$

Furthermore, the survival fraction (SF) of each group is calculated as follows:

$$\mathrm{SF}\;=\frac{\mathrm{number}\;\mathrm{of}\;\mathrm{colonies}\;\mathrm{formed}\;}{\mathrm{number}\;\mathrm{of}\;\mathrm{cells}\;\mathrm{seeded}\times \mathrm{PE}}\;$$

### 2.5 Colony Formation Assay

Colony formation assay was conducted according to the reported literature [24]. A total of 3000 TE‐1 and Kyse‐150 cells were seeded in six‐well plates, respectively. For irradiation, cells were exposed to IR at 24 h after planting and then cultured for 10−14 days. The cells were stained with Giemsa Wright solution for the assessment of colony formation. The number of colonies with >50 cells per colony was counted. Quantitative data are presented as means ± standard error of the mean (SEM) and were analyzed with the Student's *t*‐test. A *p* value of <0.05 was considered statistically significant.

### 2.6 Experimental Design for Quantitative Proteomics

To gain further understanding of radioresistance in ESCC cell lines, a TMT‐labeling quantitative proteomics study was carried out. TE‐1 and TE‐1R cells, with or without treatment, were used for the proteomic profiling. The samples were labeled using the 16‐plex Label Reagent set (Thermo Scientific, Waltham, MA, USA). TE‐1 was labeled by TMTpro‐126, 127N, and 127C; irradiated TE‐1 (TE‐1 10 Gy) was labeled by TMTpro‐128N, 128C, and 129N; TE‐1R was labeled by TMTpro‐129C, 130N, and 130C; irradiated TE‐1R (TE‐1R 10 Gy) was labeled by TMTpro‐131N, 131C, and 132N. The irradiated cells were subjected to one treatment cycle (as mentioned in Section 2.2 ).

### 2.7 Protein Extraction and Digestion for Quantitative Proteomics

Cells were harvested and lysed using urea lysis buffer containing 8 M urea (Sigma–Aldrich, St. Louis, MO, USA) and protease inhibitor cocktail (Beyotime, Cat # P1005), followed by sonication on ice. After centrifugation, the supernatant was collected, and the protein concentration was determined by the BCA protein quantification kit (Beyotime, #P0010). After reduction with dithiothreitol (DTT, Sigma–Aldrich, Cat #D0632‐10G) and subsequent alkylation with iodoacetamide (Aladdin, Cat# I105563), the lysate was diluted with 25 mM ammonium bicarbonate solution to achieve a final concentration of 1 M urea. Afterwards, trypsin was added at 1:50 trypsin‐to‐protein mass ratio for the digestion overnight. The tryptic peptides were purified by MonoSpin C18 column (GL Science, Cat# 7510‐11320) and lyophilized.

### 2.8 Peptide Labeling

Lyophilized peptides were dissolved in 100 mM TEAB (pH 8.5, Sigma–Aldrich, Cat# T7408). Add 20 µL of the TMTpro Label Reagent to each sample (see experimental design). Incubate for 1 h at room temperature. To quench the reaction, 5 µL of 5% hydroxylamine was added to the samples. Equal amounts of each sample were combined, purified, and lyophilized.

### 2.9 LC‐MS/MS Analysis

Labeled peptides were resuspended in a water solution containing 5% acetonitrile and 0.1% formic acid (FA, Fluka, Charlotte, NC, USA). Subsequently, peptides were analyzed using LTQ‐Orbitrap Elite (Thermo‐Fisher) equipped with MonoCap C18 High Resolution 3000 column (GL Science). Samples were separated by buffer A (0.1% FA) and buffer B (0.1% FA, 98% ACN). The gradient was set as follows: 5% B, 2 min; 5%–30% B, 150 min; 30%–90% B, 10 min; 90% B, 10 min; 90%–5% B, 10 min; and 5% B, 5 min. For the MS method, the spray voltage was 1.8 kV. Data‐dependent analysis (DDA) was chosen as the data acquisition mode for a higher‐quality secondary spectrum. The resolution of full mass scanning was set at 60,000, the scan range was 300–2000 *m*/*z*, and the resolution of tandem scanning was set at 15,000.

### 2.10 Data Processing

Proteome Discoverer (v2.5, Thermo‐Fisher) was employed for data processing. Variable modifications contained oxidation and acetyl (protein N‐term); fixed modifications included carbamidomethyl. The false discovery rate (FDR) cutoff value was set to 0.05.

Molecular pathways were analyzed using Ingenuity Pathway Analysis (IPA) software (Qiagen, Content version: 90348151). The *z*‐score was calculated by IPA. A positive *z*‐score predicts the activation of a pathway or function, while a negative *z*‐score suggests the inhibition.

### 2.11 Western Blot Analysis

Cells were harvested and lysed with M‐PER mammalian protein extraction reagent (Thermo scientific, Cat#WE322832), containing the protease inhibitors cocktail (Beyotime, Cat# P1005). Protein concentration was determined using the BCA protein quantification kit (Beyotime, Cat# P0012). Equal amounts of proteins were loaded to SurePAGE precast gels with a linear gradient between 4% and 20% (GenScript, Nanjing, China, Cat# M00656). Afterwards, the eBlot L1 protein transfer system (GenScript) was employed to transfer the proteins onto PVDF membranes (Millipore, Billerica, MA, USA). The membranes were then incubated with QuickBlock Blocking Buffer (Beyotime, Cat# P0239) for 1 h at room temperature and primary antibodies overnight at 4°C. After washing, the membranes were then incubated with HRP‐conjugated secondary antibodies for 1 h at room temperature. The protein bands were visualized using a ECL Western Blotting Substrate (Tanon, Shanghai, China, Cat# 180‐501). Images were collected using the Tanon‐5200 Chemiluminescent Imaging System (Tanon). The level of β‐actin was considered as the loading control.

### 2.12 RNA Interference

The negative control siRNA (NC siRNA) and siRNAs targeting the selected candidates were designed and synthesized by GenePharma (Shanghai, China). The downregulation of each candidate was achieved by utilizing two siRNAs to avoid potential off‐target effects. Transient transfection of siRNAs was performed using lipofectamine 3000 (Themo‐Fisher) following the manufacturer's instructions. The siRNA sequences are as follows:

siNC: UUCUCCGAACGUGUCACGUTT;

siTMSB10‐1:UAUCGAAGCUGGCGAUUUCTT;

siTMSB10‐2: UCAAUGGUCUCUUUGGUCGTT;

siTGM2‐1:GAAGAGCAAAAUGAAGUGGTT;

siTGM2‐2:ACAUCUAGAAGGAUCAGGCTT;

siPYGB‐1: AGAUAAUAAAUGCGCUUGGTT;

siPYGB‐2:UCAUUUGGAUACAGGACCCTT;

siS100A6‐1:UGUAGAUCAAAGCCAAGGCTT;

siS100A6‐2:UUCAGCCCUUGAGGGCUUCTT.

### 2.13 Cell Viability Assay

Cells (1000 cells per well) were plated onto 96‐well plates and incubated for 24, 48, 72, 96, and 120 h, respectively. To assess cell viability, 10 µL of Cell Counting Kit‐8 (CCK8) reagent (A311‐01 CCK8 Cell Counting Kit 500 RXNS, Vazyme Biotech Co., Ltd, Nanjing, China, Cat# A311‐01) was added into each well and allowed to incubate for 2 h. The absorbance at 450 nm was measured using a microplate reader (Thermo Scientific Multiskan MK3). The cell viability assay was repeated three times with six replicates.

### 2.14 Immunofluorescence Assay for γH2AX Foci

Transfected cells were exposed to 10 Gy x‐rays. After 24 h of irradiation, the cells were washed with cold PBS and fixed with 4% paraformaldehyde for 15 min, followed by the addition of 0.2% Triton‐100 (Solarbio, China, Cat# T8200) for cell permeabilization. Nonspecific binding was blocked by adding 5% BSA (Multi Sciences, China, Cat# A3828‐100) for 1 h. The cells were then incubated at 4°C overnight with an anti‐γH2AX antibody (ab81299, Abcam). Subsequently, the cells were incubated with a fluorescent secondary antibody (Multi Sciences, China, Cat#GAR007) for 1 h and then stained with the DAPI‐containing antifading mounting medium (Solarbio, Beijing, China Cat# S2135). Images were captured with the Confocal Microscope LSM 880 (Zeiss, Oberkochen, Germany). Fluorescence images were taken in five fields of each section at random.

### 2.15 Comet Assay for DNA Damage Evaluation

The transfected cells were irradiated with 10 Gy x‐ray radiation and then subjected to DNA damage analysis using a neutral comet assay (Comet Assay Kit, Trevigen, MN, USA, Cat# 4250‐050‐K) following the manufacturer's instructions. After washing with PBS, the cells were resuspended at a concentration of 5 × 10^5^ cells/mL, mixed with molten LMAgarose at 37°C, and immediately pipetted onto a slide using a pipette. The slides were stained with DAPI (Sigma, USA, Cat# 28718‐90‐3) after lysis buffer treatment DNA precipitation. The Comet Assay IV platform (Perceptive Instruments) was used to calculate the average tail moment for 50 cells per sample. Cells from six randomly selected fields were counted from three independent experiments.

### 2.16 HR/NHEJ Reporter Assay

U2OS‐DR‐GFP and U2OS‐EJ5‐GFP cells were transfected with candidate‐targeting siRNAs or the control siRNA in 12‐well plates. After 24 h, 1 µg pCBASceI was transfected into the U2OS reporter cells using Lipofectamine 3000. The number of GFP^+^ cells was measured 48 h posttransfection using a FACSCanto II flow cytometer (BD Bioscience, CA, USA). Each experiment included three replicated wells and was repeated three times.

### 2.17 Immunohistochemistry Staining

Paraffin‐embedded tissue sections were deparaffinized and heat‐treated with citrate buffer, pH 6.0, for 5 min for epitope retrieval. To prevent endogenous peroxidase activity and nonspecific antibody binding, sections sequentially treated with 0.03% hydrogen peroxide for 10 min and 5% BSA for 1 h at room temperature. Subsequently, the sections were incubated with the primary antibody overnight at 4°C. After washing with PBS, HRP‐conjugated secondary antibody was applied, and the sections were incubated for 1 h at room temperature. Then, the sections were prepared by adding 3‐3ʹ‐diaminobenzidine (DAB) (Cwbio, China, Cat# P10100) and were subsequently counterstained with hematoxylin. The stained sections were analyzed and evaluated using a Leica Microscope (Leica, Wetzlar, Germany). Evaluation was based on the intensity and average percentage of positive cells. The staining intensity was categorized as: “1” (negative or weakly positive), “2” (moderately positive), and “3” (strongly positive). The average percentage of positive cells was rated as 1 (<25%), 2 (25%−50%), 3 (50%−75%), and 4 (>75%).

### 2.18 Statistical Analysis

All data were presented as the mean ± SEM from a minimum of three independent experiments. Differences among groups were evaluated with Student's *t*‐test (paired *t*‐test or two‐sample *t*‐test) or one‐way analysis of variance (ANOVA) analysis followed by subsequent Dunnett's multiple comparisons test. Graphs were generated using Graph Prism 8 software. A *p* value < 0.05 was considered statistically significant. Cell‐based experiments were performed in technical triplicates and with each group measured in three biological replicates (experimental replicates). No randomization or blinding procedures were carried out in this study.

## 3 Results

### 3.1 Proteomic Profiling of ESCC Radioresistant Cells

**FIGURE 1:**
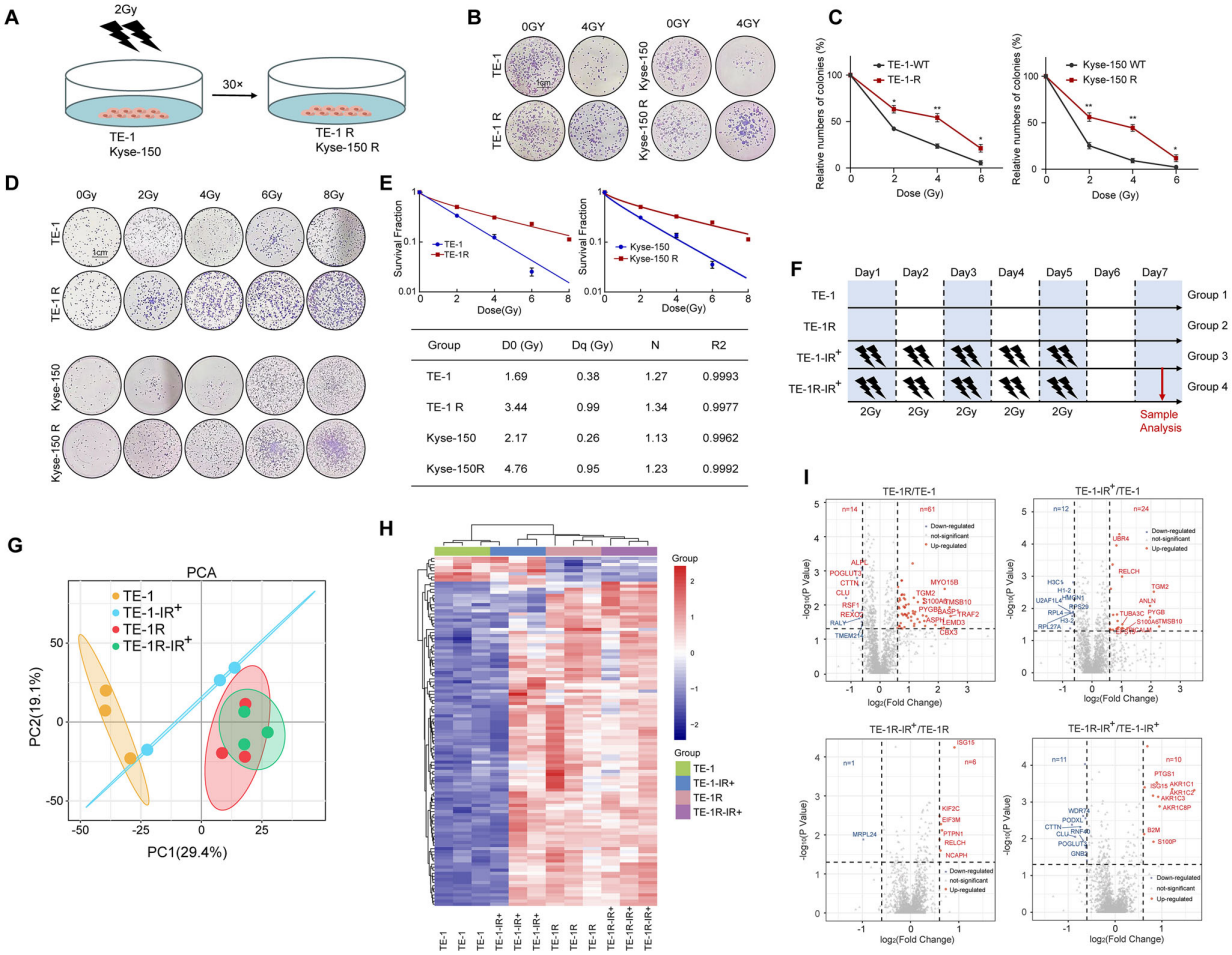
Proteomic profiling of ESCC radioresistant cells. (A) Construction of radioresistant cell line in esophageal squamous cell carcinoma. (B) Cell colony formation assays of radioresistant cells and their parental cells. Six‐well plates containing TE‐1 and TE‐1 R, Kyse‐150, and Kyse‐150R cells were planted with 3000 cells per well and were exposed to IR at 0–6 Gy. (C) Relative numbers of colonies. Data are means ± SEM from three independent experiments. *p < 0.05, **p < 0.01 versus corresponding value for control groups (Student's *t*‐test). (D) Clonogenic survival assay of cells after irradiation. Cells were seeded in six‐well plates at different 200, 1000, 2000, 6000, and 12,000 per well. The cells were irradiated with doses of 0, 2, 4, 6, and 8 Gy, respectively. (E) A multitarget single‐hitting model was used to fit cells to a survival curve. The *k*, *N*, *D*_0_, and *D*_q_ value were fitted by GraphPad software. (F) Time line for multifractionated irradiation with TE‐1 cells. (G) Bidimensional principal component analysis (PCA) of proteomic profiles in TE‐1 cells. (H) Heatmap of expression profiles for differential proteins. (I) Volcano plot of differential proteins in four groups. ESCC, esophageal squamous cell carcinoma; IR, ionizing radiation; SEM, standard error of the mean.

In our previous study, we have established a radioresistant ESCC cell line, TE‐1R, derived from its parental cell line TE‐1 [22]. In order to further explore the mechanism of RT resistance in ESCC cells, we employed a similar approach to create an RT‐resistant cell line, Kyse‐150R, using the wild‐type parent cell line Kyse‐150 as a basis (Figure 1A). A clonogenic cell survival assay was conducted to evaluate their radiosensitivity. Both TE‐1R and Kyse‐150R showed a greater number of colonies compared to the wild‐type controls (Figure 1B,C). Additionally, the results in Figure 1D confirmed that the radioresistant cell lines exhibited significantly increased resistance to IR compared to the parent cell lines in terms of cell survival. This is supported by the single‐hit multitarget model, as the TE‐1R and Kyse‐150 R cells showed higher SFs than their parent cells. Moreover, both *D*_q_ and *D*_0_ were increased in radioresistant cells, indicating increased sensitivity to IR in TE‐1 R and Kyse‐150 R cells (Figure 1E).

To investigate the potential protein factors associated with the development of acquired radioresistance in ESCC cells, we divided the cells into four distinct groups (Figure 1F): Group 1 = wild‐type cells without IR as the control group (TE‐1), Group 2 = IR‐resistant cells without IR (TE‐1R), Group 3 = wild‐type cells exposed to IR treatment (TE‐1‐IR^+^), and Group 4 = IR‐resistant cells following IR exposure (TE‐1R‐IR^+^). The IR exposure cells involved daily doses of 2 Gy x‐ray radiation for five consecutive days, followed by a 2‐day break. Subsequently, a quantitative proteomic study using a tandem mass tag (TMT) was conducted, leading to the identification of 2439 proteins with an FDR <1%. The protein expression patterns, as assessed by principal component analysis (PCA) among all groups, are presented in Figure 1G, providing a comprehensive overview of our findings. The expression patterns of the identified proteins (with fold change [FC] ± 1.5, FDR < 0.05) in the samples shown in Figure 1H were analyzed using hierarchical clustering, revealing significant differences between TE‐1 and TE‐1R cells. The results indicated that, in the absence of radiation treatment, the protein expression patterns of radiosensitive and radioresistant cells are distinct. However, after radiation exposure, the differences in protein expression signatures between radiosensitive and radioresistant cells become less apparent. Specifically, the protein expression profiles of radiosensitive cells before and after IR treatment form two distinct groups, while the protein expression profiles of radioresistant cells show minimal differences before and after radiation. Furthermore, in the hierarchical clustering analysis, one sample in the TE‐1‐IR+ conditions deviated from the norm as it grouped with TE‐1 samples, aligning with the findings of the PCA analysis in Figure 1G. Hence, we suspect the presence of an outlier in the TE‐1‐IR+ group, potentially stemming from individual variances among cell samples from different batches.

Further analysis is focused on paired comparisons, as illustrated in Figure 1I. The selection of differentially expressed proteins was based on the following criteria: upregulated proteins with an FC > 1.5 and *p* < 0.05, while downregulated proteins with FC < 0.67 and *p* < 0.05. In comparison to the parent radiosensitive TE‐1 cell line, 61 proteins were significantly upregulated, and 14 proteins were significantly downregulated in the radioresistant TE‐1R cell line. It is evident that 24 proteins were upregulated and 12 proteins were downregulated in TE‐1 cells following multiple radiation treatments, compared to the non‐treated control group. However, the changes observed in TE‐1R cells before and after IR are minimal, with only six upregulated and one downregulated proteins identified. Additionally, fewer differentially expressed proteins were found between the two cell lines following IR, including 10 upregulated and 11 downregulated proteins.

These data suggested that, in the absence of IR treatment, significant disparities in protein expression were observed between cells displaying acquired radioresistance and those exhibiting radiosensitivity. Following irradiation, wild‐type (radiosensitive) cells exhibited alterations in protein expression, whereas the changes in protein expression in radioresistant cells following RT were minimal.

### 3.2 Bioinformatic Analysis of Differentiated Proteins

**FIGURE 2:**
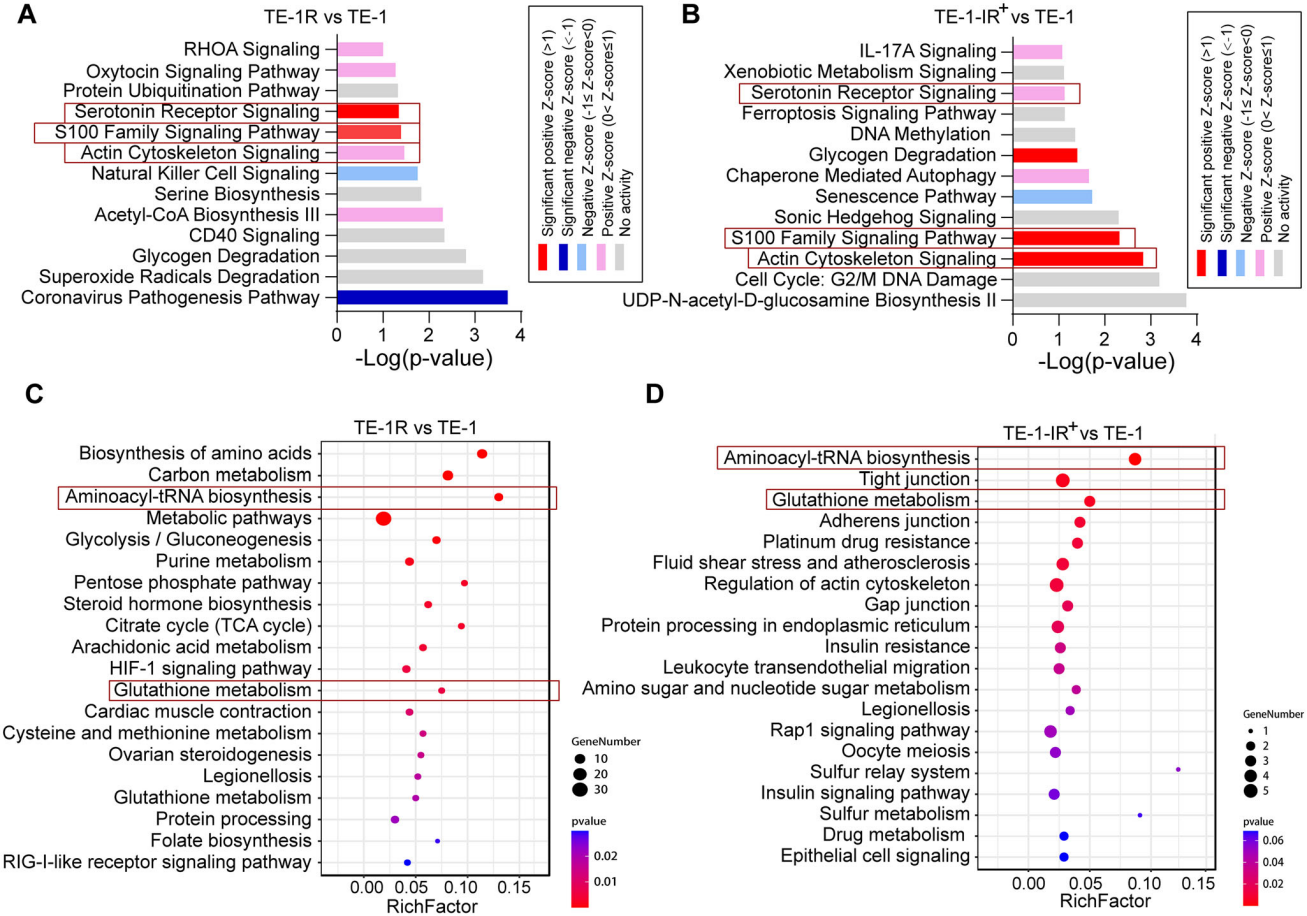
Bioinformatic analysis of differentiated proteins. (A, B) IPA pathway analysis of differentially upregulated genes. (C, D) KEGG pathway analysis of upregulated genes. IPA, Ingenuity Pathway Analysis; KEGG, Kyoto Encyclopedia of Genes and Genomes.

The differential expression of proteins was analyzed using IPA (Qiagen, http://www.qiagen.comingenuity). In Figure 2A, the enrichment of key activated pathways, including S100 Family Signaling Pathway, Actin Cytoskeleton Signaling, and Serotonin Receptor Signaling pathways, was demonstrated between radioresistant and radiosensitive cells. Interestingly, these pathways were also enriched in wild‐type TE‐1 cells exposed to 10 Gy fractionated IR, highlighting their essential role in mediating the cellular response to IR treatment (Figure 2B).

The KEGG pathway analysis revealed enrichment of upregulated proteins in aminoacyl‐tRNA biosynthesis, glutathione metabolism, and several metabolic pathways in radioresistant cells without RT treatment and in radiosensitive cells treated with a total10 Gy fractionated radiation doses. A comparison of the two groups of upregulated proteins showed an intersection between tRNA assembly and S100 family (Figure 2C,D).

### 3.3 Validation of the Candidates in Response to IR

**FIGURE 3:**
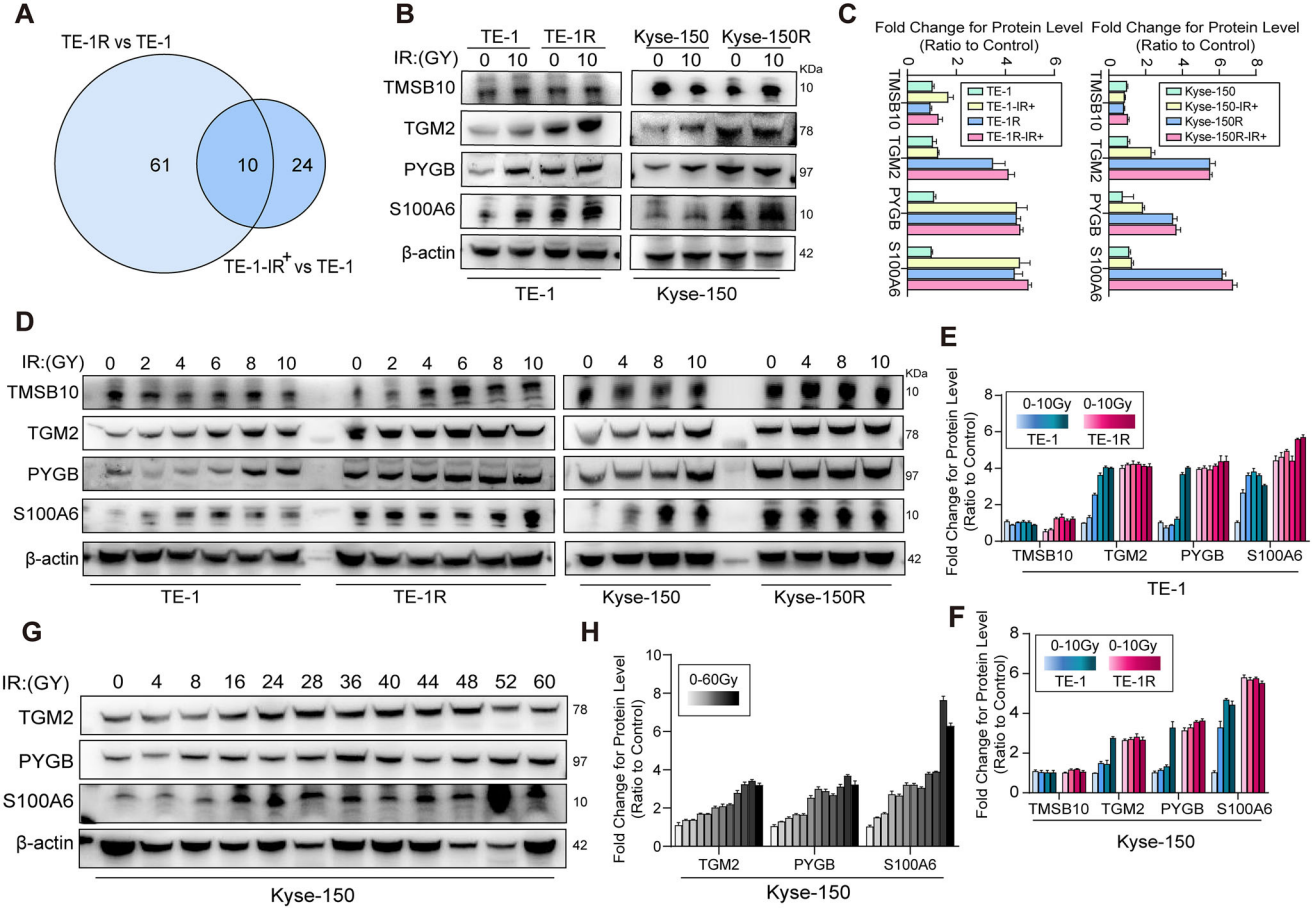
Validation of the candidates in response to IR. (A) Venn plot shows the number of intersection genes between two groups: TE‐1R versus TE‐1 and TE‐1 10 Gy versus TE‐1 0 Gy. (B, C) The protein level of four candidate targets was detected by Western blot analysis in TE‐1 and Kyse‐150 cells (IR‐treated and untreated groups). The proteins were obtained 24 h after IR and the IR cumulative dose of 10 Gy. Protein levels were normalized to the untreated group. (D–F) WB analysis of protein expression in four candidate genes multifractionated irradiation treated TE‐1 and Kyse‐150 cells. TE‐1 and Kyse‐150 cells were collected for extract protein samples for Western blot analysis 24 h after receiving 2, 4, 6, 8, and 10 Gy radiation doses to cells. Results are expressed as the fold increase relative to the untreated control cells within each treatment group. (G, H) Representative immunoblots of TGM2, PYGB, and S100A6 levels were determined by Western blotting. Kyse‐150 cells received an accumulated irradiation dose of 60 Gy and during the radiotherapy process, some cells were collected for protein detection during each passage of cells. Untreated parental Kyse‐150 cells are used as a control. IR, ionizing radiation; PYGB, glycogen phosphorylase, brain form; S100A6, S100 calcium‐binding protein A6; TGM2, glutamine gamma‐glutamyltransferase 2.

After identifying the activated pathways, we shifted our focus to the proteins that were upregulated for further examination. Upon comparison of the proteins in the two groups, we identified 10 targets that were commonly found, as indicated in the Venn diagram (Figure 3A) and Table 1. The selected candidate genes were chosen based on differential protein expression levels and their enrichment in pathways that may impact tumor RT. The genes selected for this study are: thymosin beta‐10 (TMSB10), protein‐glutamine gamma‐glutamyltransferase 2 (TGM2), glycogen phosphorylase, brain form (PYGB), and protein S100 calcium‐binding protein A6 (S100A6). These candidates are associated with cell motility [25, 26], cancer metabolism [6, 27], and tumor cell transport [28], all of which have been implicated in tumor radio and drug resistance.

**TABLE 1:** The radioresistant‐regulated proteins in esophageal squamous cell carcinoma (ESCC) cells.

Gene symbols	Description	Biological process
TMSB10	Thymosin beta‐10	Sequestering of actin monomer
PYGB	Glycogen phosphorylase, brain form	Cellular carbohydrate metabolic
TGM2	Protein‐glutamine gamma‐glutamyltransferase 2	Primary metabolic process
S100A6	Protein S100‐A6	Transport and cellular process
EPS15	Epidermal growth factor receptor substrate 15	Receptor‐mediated endocytosis
HARS1	Histidine–tRNA ligase	Peptide metabolic process
ADK	Adenosine kinase	AMP biosynthetic process
RRBP1	Ribosome‐binding protein 1	Peptide metabolic process
TPM4	Tropomyosin alpha‐4 chain	Cellular component or biogenesis
HSPA14	Heat shock 70 kDa protein 14	Protein refolding

We assessed the protein levels of the four potential candidates in the original radiosensitive and radioresistant cells. Our findings revealed that the radioresistant cells exhibited significantly higher levels of TGM2, PYGB, and S100A6 compared to their original counterparts in both models (Figure S1A). However, we did not observe any changes in the TMSB10 protein level. Additionally, the expression of TGM2, PYGB, and S100A6 was found to be increased in both radiosensitive and radioresistant cells after exposure to fractionated IR, whereas no similar protein overexpression pattern of TMSB10 was observed (Figure 3B,C). To further monitor dynamic changes in the protein levels of these four candidates during treatment, we obtained protein samples from the two radiation‐resistant cell lines and their parental cell lines, which underwent radiation doses ranging from 0 to 10 Gy. As shown in Figure 3D–F, protein levels of TGM2, PYGB, and S100A6 increased progressively with accumulated doses of IR, while the protein level of TMSB10 remained constant in both TE‐1 and Kyse‐150 cells. Furthermore, consistent with our proteomic results, fractionated IR did not induce the upregulation of TGM2, PYGB, and S100A6 in the radioresistant cell lines, and these protein levels remained elevated. To further validate these protein level changes and their relationship with the development of acquired radioresistance in ESCC cells, we analyzed protein samples during the establishment of the Kyse‐150 cell line. Our observations revealed a gradual increase in the expression levels of TGM2, PYGB, and S100A6 as they were exposed to radiation doses ranging from 0 to 60 GY, demonstrating a dose‐dependent response (Figure 3G,H).

### 3.4 Downregulation of Candidates Sensitized Radiation Refractory ESCC Cells

**FIGURE 4:**
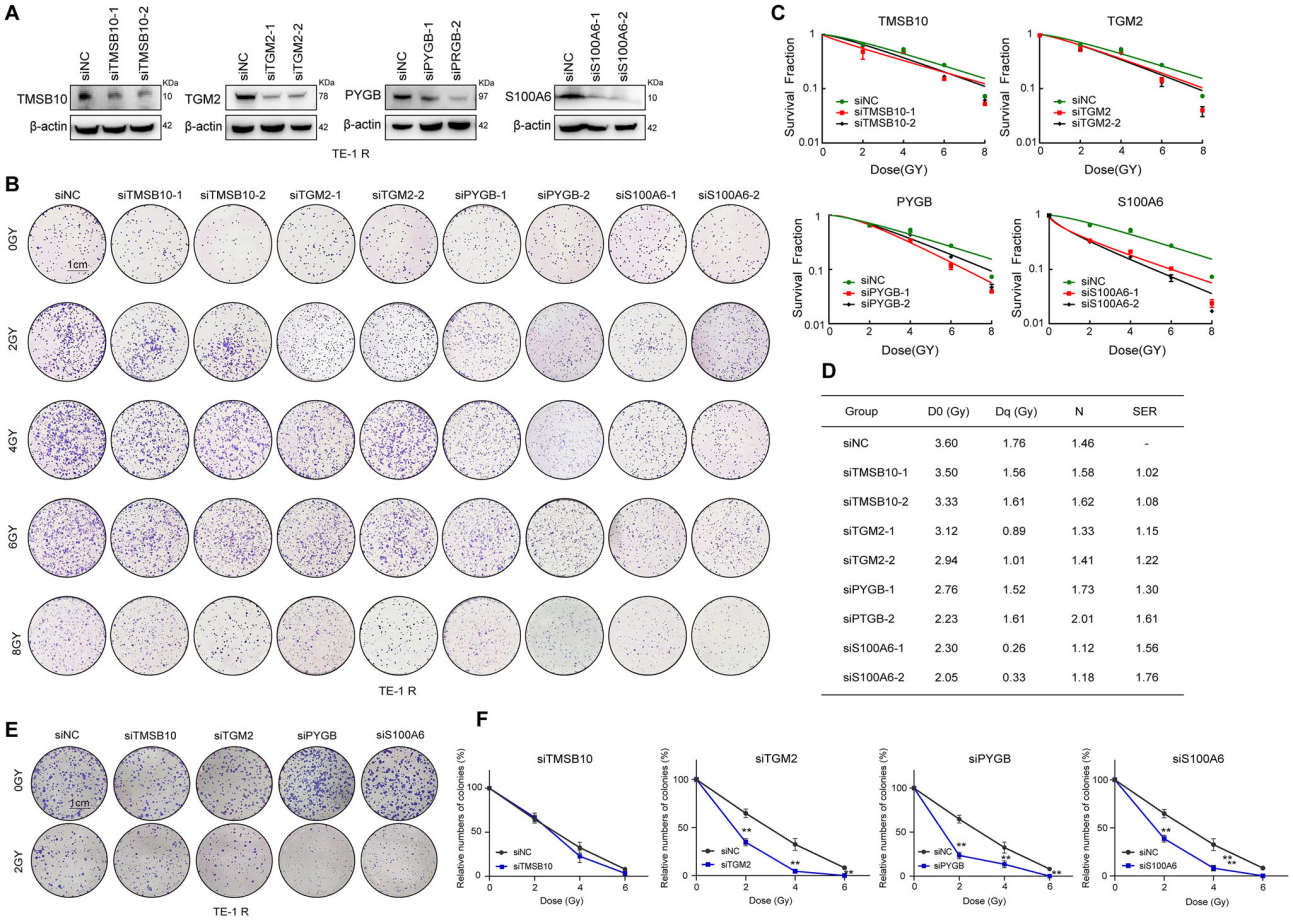
Downregulation of candidates sensitized radiation refractory ESCC cells. (A) Immunoblotting for TMSB10, TGM2, PYGB, and S100A6 in TE‐1R cells after transfection with siRNA. (B) Clonogenic survival assay of cells after irradiation to measure the impact of TMSB10, TGM2, PYGB, and S100A6 siRNAs in ESCC cells. Transfected TE‐1R cells were seeded at clonal density (200, 1000, 2000, 6000, and 12,000 per well) and then performed with radiation treatment with 0, 2, 4, and 8 Gy, respectively (*n* = 3). (C, D) The single‐hit multitarget model was used to establish cell survival curves after ionizing radiation. The *k*, *N*, *D*_0_, and *D*_q_ values were fitted by GraphPad software. (E, F) A clone formation assay was performed to measure the relative colony numbers of each experimental group. Transfected TE‐1R cells were seeded in six‐well plates at 3000 per well, and radiotherapy was performed in different dose groups. Data are means ± SEM from three independent experiments. *p < 0.05, **p < 0.01 versus corresponding value for control groups (Student's *t*‐test). ESCC, esophageal squamous cell carcinoma; PYGB, glycogen phosphorylase, brain form; S100A6, S100 calcium‐binding protein A6; SEM, standard error of the mean; TGM2, glutamine gamma‐glutamyltransferase 2; TMSB10, thymosin beta‐10.

In order to investigate the functional connections between the four candidates and radioresistance, we employed siRNA knockdown to reduce the expression of TMSB10, TGM2, PYGB, and S100A6 (Figure 4A and Figure S1B). Following this, clonogenic survival assays were conducted on radiation‐resistant TE‐1R cells, exposing them to various single radiation doses. As shown in Figure 4B,C, downregulation of TGM2, PYGB, and S100A6 led to decreased colony formation and SF, whereas TMSB10‐knockdown did not affect ESCC cell colony formation after radiation. In comparison to the control group, the *D*_q_ and *D*_0_ values decreased, and the sensitization ratio increased after TGM2, PYGB, and S100A6 siRNA knockdown and combined irradiation treatment, while radiosensitivity did not improve in the TMSB10 knockdown combined RT group (Figure 4D). Subsequently, we performed colony formation assays in TE‐1 R cells exposed to a series of single radiation doses (Figure 4E,F). Knockdown of TGM2, PYGB, and S100A6 reduced the colony formation ability in vitro after IR, while TMSB10 knockdown did not result in diminished in response to IR. Consistent with these findings, radioresistant TE‐1R cells with TGM2, PYGB, and S100A6 knockdown were significantly more sensitive to IR compared to the control cells, underscoring the significance of TGM2, PYGB, and S100A6 for radioresistance in ESCC cells.

Remarkably, S100A6 siRNAs exhibited the most potent sensitization effect. After 48 h of radiation therapy, there were no significant differences in cell apoptosis rates between the downregulated groups of TMSB10, TGM2, and S100A6 compared to the control group, while there was a significant difference between the downregulated PYGB group and the control group (Figure S1C, D).

### 3.5 Knockdown of Candidates Exacerbated DNA Damage After Fractionated IR

**FIGURE 5:**
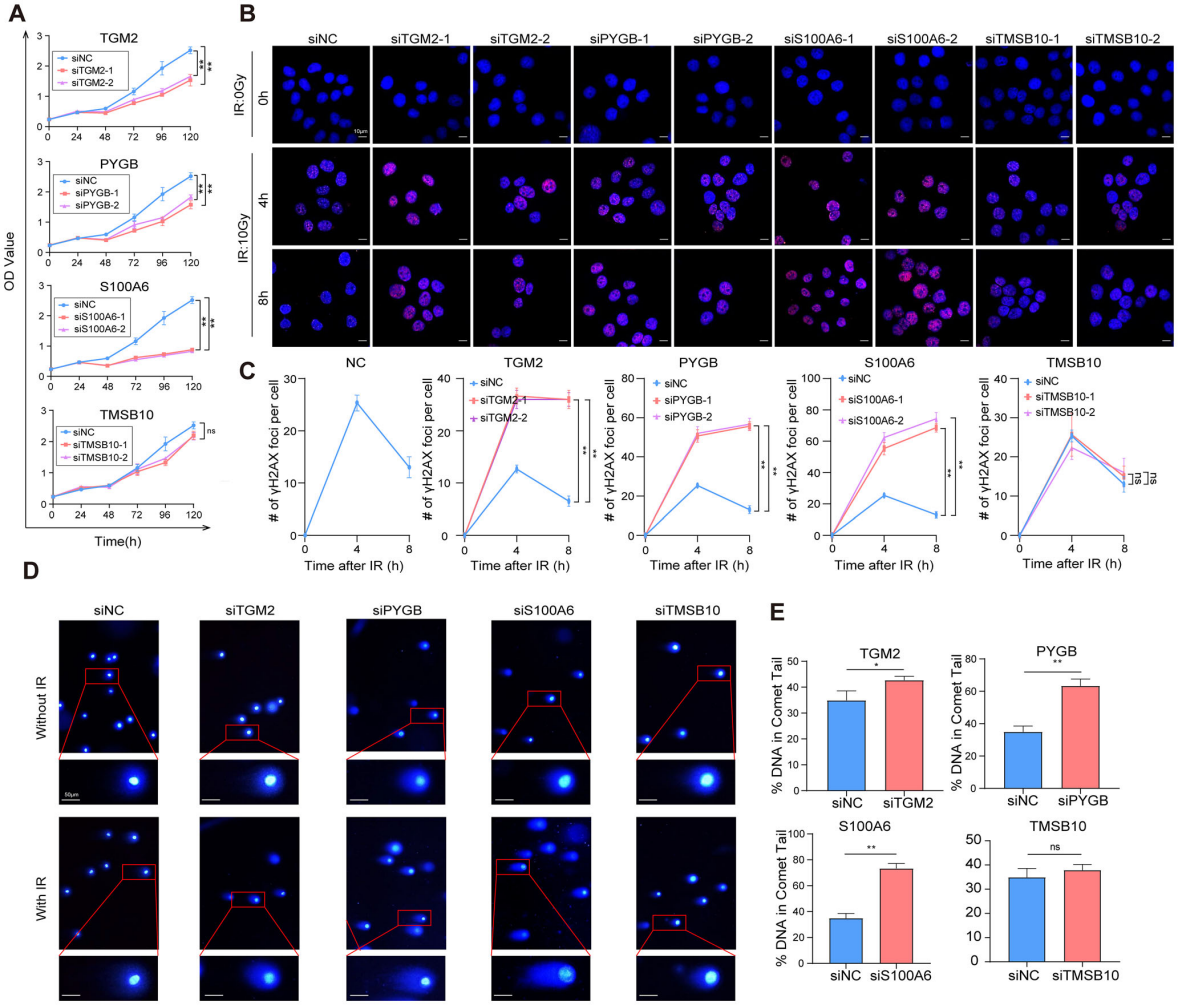
Knockdown of candidates exacerbated DNA damage after fractionated IR. (A) CCK8 analysis was performed to assess the viability of TE‐1R cells transfected with four siRNAs, followed by treatment with 10 Gy IR. (B, C) Representative images of γH2AX‐positive nuclei in TE‐1R cells transfected with four siRNAs 24 h post‐IR. The 0 h means no IR. γH2AX foci in red, nuclear counterstaining with 4′,6‐diamidino‐2‐phenylindole in blue. Scale bar: 10 µm. *p < 0.05; **p < 0.01. (D, E) Comet assay was carried out in TE‐1R cells transfected with four siRNAs at the indicated time points after IR treatment. Scale bar: 50 µm. *p < 0.05; **p < 0.01. CCK8, Cell Counting Kit‐8; IR, ionizing radiation.

Similar results were noted in the cell viability assay using CCK8. Following downregulation, cells were exposed to fractionated IR and reseeded in 96‐well plates. Figure 5A illustrates that the reduction of TGM2, PYGB, and S100A6 by siRNAs resulted in reduced viability compared to the control group. The presence of γ‐H2AX foci serves as a reliable marker of DNA double‐strand breaks (DSBs), indicating the occurrence of DNA damage and reflecting the DNA repair capacity and radiosensitivity. Therefore, we assessed the level of γ‐H2AX foci in ESCC cells after IR to investigate the influence of these factors on DNA damage. As depicted in Figure 5B,C, depletion of TGM2, PYGB, and S100A6 in TE‐1 cells led to an increased number of γ‐H2AX foci compared to the control group at 4 and 8 h after IR stimulation, suggesting more severe IR‐induced damage and impaired DNA damage repair (DDR) process. In contrast, TE‐1R siTMSB10 cells showed fewer γ‐H2AX foci following IR, and no foci were observed in the absence of IR (Figure S2A).

Furthermore, a comet assay was conducted to investigate the DSBs following fractionated IR. Consistent with the findings of γ‐H2AX foci, it was observed that the comet tails were elevated in the TGM2, PYGB, and S100A6 deficient cells compared to the control cells and the cells without IR, while TMSB10 knockdown resulted in significantly shorter comet tails (Figure 5D,E).

These results suggested that decreased levels of TGM2, PYGB, and S100A6 sensitized radioresistant ESCC cells to IR treatment and may play a crucial role in the development of acquired radioresistance.

### 3.6 S100A6, TGM2, and PYGB Regulate DSB Repair Through Homologous Recombination (HR) Repair Pathway or Nonhomologous End Joining (NHEJ) Repair Pathway While S100A6 Was Correlated With ESCC Recurrence

**FIGURE 6:**
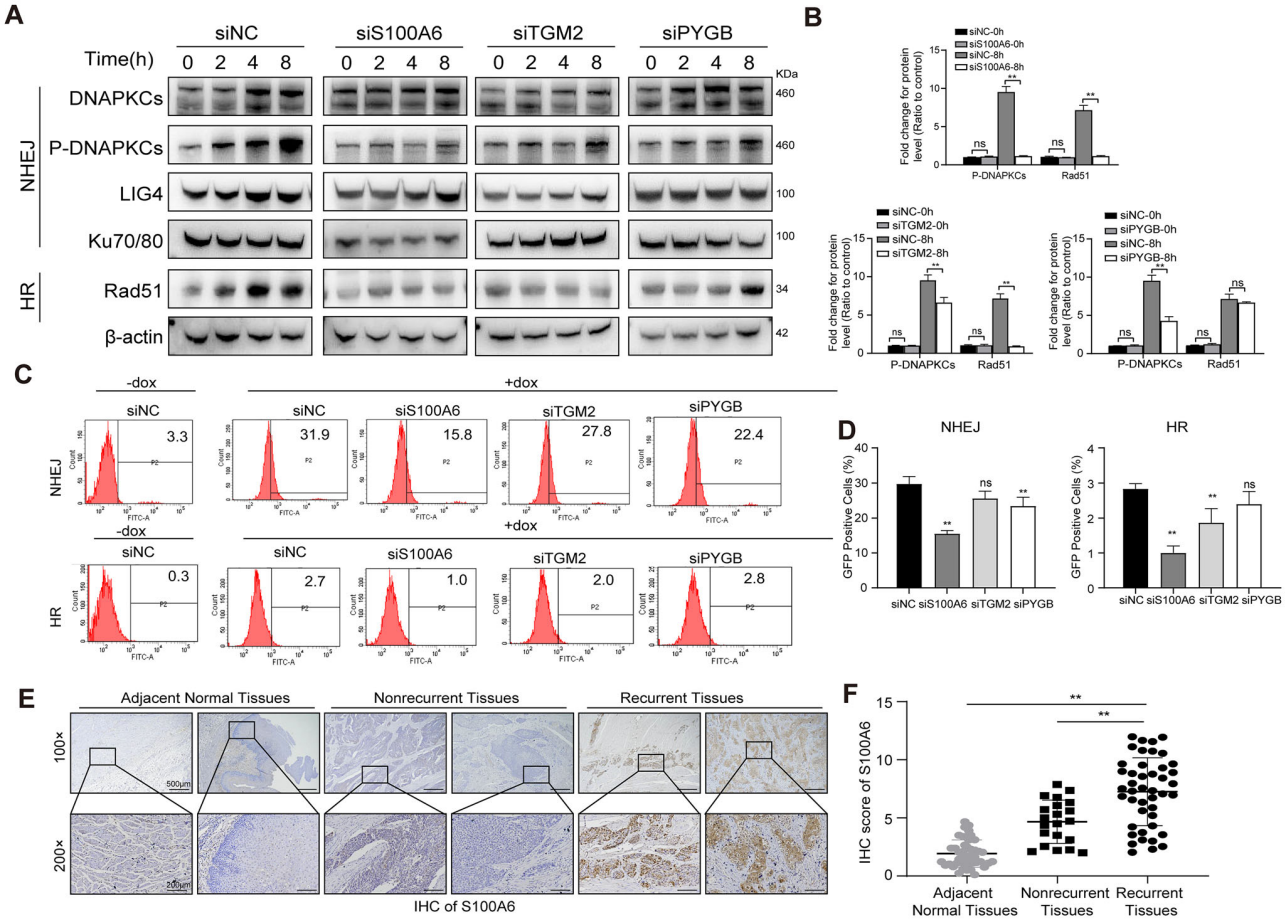
S100A6, TGM2, and PYGB regulate DSB repair through homologous recombination (HR) repair pathway and nonhomologous end joining (NHEJ) repair pathway, while S100A6 was correlated with ESCC recurrence. (A) WB analysis of HR and NHEJ‐related proteins influenced by S100A6, TGM2, and PYGB expression following 10 Gy IR at different time points. (B) The protein intensity of DNAPKCs and Rad51 was quantified with ImageJ. (C, D) Detection of DNA repair pathways using flow cytometry in U2OS cells transfected with S100A6, TGM2, and PYGB siRNAs at the indicated time points after IR treatment. (E) Immunohistochemical analysis was conducted by using ESCC tissues as primary and recurrence tumors. DSB, DNA double‐strand break; ESCC, esophageal squamous cell carcinoma; PYGB, glycogen phosphorylase, brain form; S100A6, S100 calcium‐binding protein A6; TGM2, glutamine gamma‐glutamyltransferase 2.

It has been reported that HR and NHEJ are the two critical pathways involved in DDR. Thus, we investigated the expression levels of essential proteins involved in HR and NHEJ in ESCC cells to evaluate their impact of those candidates on these pathways. As shown in Figure 6A,B, the TE‐1R‐siS100A6 and siPYGB groups exhibited reduced expression of Ku70/80 and decreased DNA‐PKcs phosphorylation after IR, indicating their crucial roles as NHEJ effector proteins. Moreover, TGM2 and S100A6 influenced the expression levels of Rad51, suggesting that PYGB and S100A6 also regulate the HR pathway. After 24 h of radiation therapy, the protein level of p‐DNAPKCs in the TGM2 knockdown group was partially downregulated compared to the NC group, while the level of Ku70/80 did not show significant changes.

As the candidates were suppressed, which impacts crucial effector proteins in the two main DSBs repair pathways, it is possible that their ability to repair could be compromised. Therefore, a flow cytometry‐based analysis was carried out to evaluate the repair efficiency of the HR and NHEJ pathways in the radioresistant cells after siRNA interference. In consistent with the results from immunoblotting, S100A6 was observed to simultaneously impact the repair abilities of both HR and NHEJ pathways in the radioresistant cells. Knockdown of TGM2 disrupted the HR pathway, while downregulation of PYGB reduced the radiosensitivity of ESCC cells by influencing the NHEJ pathway (Figure 6C,D).

These findings collectively suggest that S100A6 may simultaneously promote the repair of IR‐induced DNA damage through both NHEJ and HR pathways, while TGM2 controls NHEJ and PYGB influences the HR pathway, thus leading to the development of radioresistance in ESCC cells. It is worth noting that S100A6 exhibited the most significant sensitization effect among all candidates in radioresistant ESCC cells.

To further validate the expression levels of S100A6 in ESCC patient samples, immunohistochemistry (IHC) analysis was performed on ESCC tissues with or without recurrence and their matched adjacent nontumor tissues. Our findings demonstrated a significant upregulation of S100A6 expression in patients with recurrent ESCC compared to those without recurrence, as well as in the adjacent nontumor tissues (Figure 6E). However, there was no correlation between the expression levels of S100A6 and gender, age, T stage, N stage, or pathological stage (Table 2).

**TABLE 2:** The correlation between S100A6 expression and ESCC patients’ pathological features.

	S100A6 expression		
Characteristics	Low	High	*p* value^*^
Gender			0.6300
Male	20	24	
Female	7	11	
Age			0.1483
≤60	13	14	
>60	14	31	
T stage			0.0600
T0–T1	14	10	
T2–T3	13	25	
N stage			0.0631
N0	14	16	
N1–N3	13	19	
Pathological stage			0.3138
I	7	6	
II	14	15	
III–IV	6	14	

Abbreviations: ESCC, esophageal squamous cell carcinoma; S100A6, S100 calcium‐binding protein A6.

Chi‐square test.

^*^ *p* < 0.05.

## 4 Discussion

In spite of the application of advanced surgical techniques and various adjuvant RT, chemotherapy, or chemoradiotherapy, the prognosis for patients with ESCC remains unsatisfactory [4, 5, 29]. Therefore, there is an urgent need to advance research in discovering innovative cancer diagnosis and treatment strategies. Recently, there has been widespread interest in identifying important ESCC‐related biomarkers or therapeutic targets through proteomic techniques [15, 16, 30]. In this study, we conducted quantitative proteomics to identify differently expressed proteins between radiation‐resistant and radiation‐sensitive cells in ESCC. Our findings indicate that a group of proteins in radioresistant cells exhibit elevated expression levels compared to radiosensitive cells (wild type). Interestingly, during the stimulation of clinical treatment processes, there was a gradual increase in the expression of these proteins in wild‐type cells, indicating their involvement in acquired radioresistance. In contrast, radio‐insensitive cells treated with fractionated IR consistently maintain high basal expression levels of these proteins. Subsequent functional experiments have demonstrated the critical role of TGM2, PYGB, and S100A6 proteins in maintaining radioresistance in ESCC cells. Further investigations have substantiated that S100A6, TGM2, and PYGB serve as promising biomarkers and potential therapeutic targets for radioresistance in ESCC cells.

Among these identified targets, S100A6, a member of the calcium‐binding protein family, is found to be expressed in certain tumor cells [31, 32]. Our results are consistent with previous findings, showing that S100A6 is overexpressed post‐IR in other cancer cells [33]. Nevertheless, its role in ESCC is not yet understood. Our study reveals that inhibition of S100A6 can significantly reduce the DDR capability of radiation‐resistant cells in esophageal cancer, while also suppressing the NHEJ and HR repair pathways. Interestingly, immunohistochemical analysis reveals a high expression of S100A6 in recurrent tissues compared to nonrecurrent tissues in ESCC clinical patient samples. Therefore, in ESCC, the expression of S100A6 may serve as a predictive indicator for recurrence and offer therapeutic potential.

In addition, we have identified the potential function of transglutaminase 2 (TGM2) in imparting radiation resistance to ESCC cells. TGM2 is a crucial enzyme involved in various cellular processes [34, 35, 36]. Previous research has shown that in glioblastoma, TGM2 enhances radioresistance by promoting the fusion of autophagosomes and lysosomes through its interaction with SDC1 [37]. However, its role in ESCC has been insufficiently explored. Our findings suggested that reducing TGM2 expression increased ESCC cell radiosensitivity and inhibited proliferation after RT by suppressing HR repair, indicating that it may represent a promising target for sensitization in the clinical management of ESCC.

Moreover, we have observed an increase in PYGB expression during radiation therapy for ESCC. Glycogen phosphorylase (PYGB), an enzyme involved in glycogen metabolism [38, 39], has been implicated in various tumorigenic processes, including proliferation, metastasis, and epithelial‐mesenchymal transition in breast, ovarian, and gastric cancer [38, 40, 41]. However, its role in ESCC remains uncertain. Our study demonstrates that PYGB regulates the sensitivity of ESCC cells to radiation by enhancing the NHEJ pathway. Furthermore, knocking down PYGB promotes apoptosis of ESCC cells after RT, suggesting that PYGB may represent a potential therapeutic target for treating ESCC.

An interesting finding of this study is that all previously mentioned candidates modulate NHEJ or HR, which are the two main pathways for DNA double‐strand repair breaks [42, 43, 44]. The molecular mechanisms responsible for the development of RT resistance in tumor cells are multifaceted, involving aberrant DNA repair mechanisms [42, 45], cell cycle regulation, the tumor microenvironment [46, 47], and the presence of tumor stem cells [48]. Increased DNA repair capacity is a hallmark of RT resistance, and there is mounting evidence indicating that DNA repair contributes to cancer therapy resistance [29, 42, 49]. Previous studies have indicated the reduced efficacy of IR in ESCC in the presence of hyperactive HR and NHEJ markers [6]. In this study, we found that targeted inhibition of protein expression of S100A6, TGM2, and PYGB enhances DNA damage levels in ESCC cells following RT and reduces the DNA repair capacity of ESCC cells, ultimately leading to increased radiosensitivity of ESCC cells. These proteins have diverse mechanisms that impact DNA repair, with S100A6 promoting both NHEJ and HR repair pathways in ESCC cells, while TGM2 and PYGB promote DNA repair through their respective effects on NHEJ and HR pathways.

However, there are several limitations to this study. Firstly, our focus was restricted to a specific subtype of esophageal cancer, and the general applicability of our findings requires additional validation. Secondly, as we conducted in vitro cell experiments, it is essential to expand our research to in vivo experiments and more clinical practice for further investigation. Finally, the exact mechanism underlying the modulation of these candidates in DNA repair pathways still remains unclear and demands further investigation.

In conclusion, we have initially observed disparities in protein expression profiles between cells sensitive to radiation and those resistant to it. This not only advances our understanding of the cellular mechanisms involved in the response to radiation therapy but also offers fresh perspectives for clinical intervention. Thus, our investigation uncovers potential mechanisms of resistance to RT in ESCC cells and presents opportunities for enhancing radiosensitization strategies for clinical treatment. Specific targets and their associated signaling pathways can be modulated to improve the therapeutic effectiveness in ESCC.

## Author Contributions

Study conception and experimental design: Jundong Zhou, Mu Wang. Clinical samples collection and data analysis: Ya Pan. Experimental conduction: Aidi Gao, Chao He, Hengrui Chen, Qianlin Liu, Yin Chen, and Jianyin Sun. Manuscript writing: Aidi Gao and Chao He. Bioinformatic analysis: Hengrui Chen. Radiotherapy operation: Chuanfeng Wu. Study conception: design and supervision. Sonia Rocha. Data analysis: Qianlin Liu and Ya Pan.

## Ethics Statement

The procedures of this study were approved by the Research Ethics Committee of The Affiliated Suzhou Hospital of Nanjing Medical University.

## Conflicts of Interest

The authors declare no conflicts of interest.

## Supporting information



Supporting Information

Supporting Information

## Data Availability

The protein mass spectrometry data from this paper have been archived in the iProX database (iprox.org) and given the ID IPX0008007000.
