# Supplementary figures and images for: TMT‐Based Quantitative Proteomic Profiling of Human Esophageal Cancer Cells Reveals the Potential Mechanism and Potential Therapeutic Targets Associated With Radioresistance
Source: Proteomics Clin Appl. 2024 Oct 7;19(1):e202400010. doi: 10.1002/prca.202400010 (PMC11726268; doi:10.1002/prca.202400010)

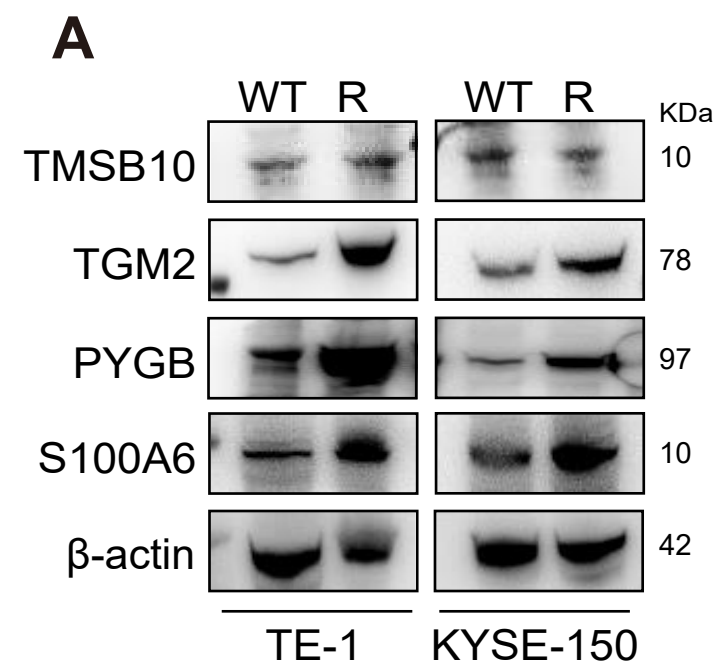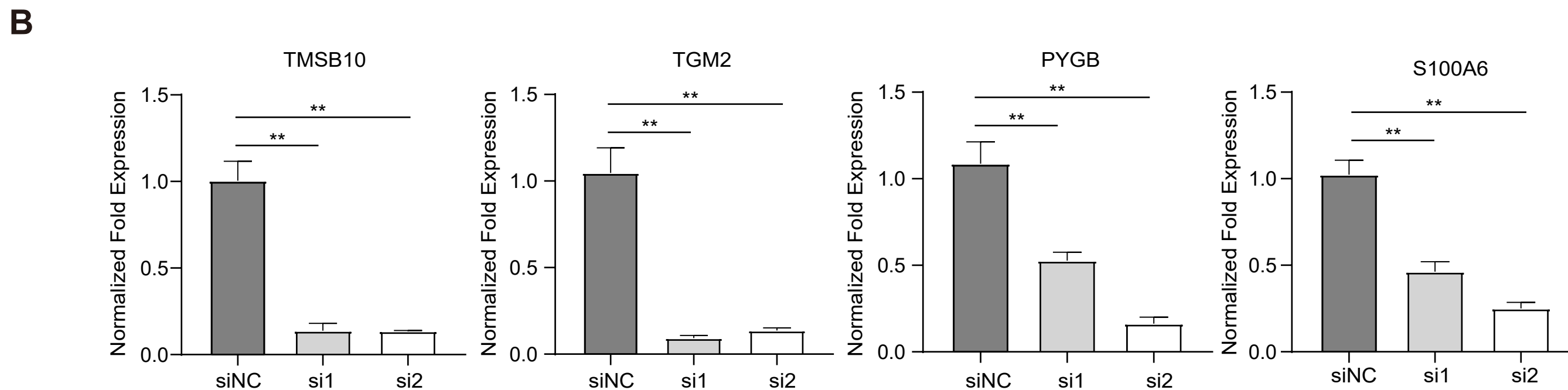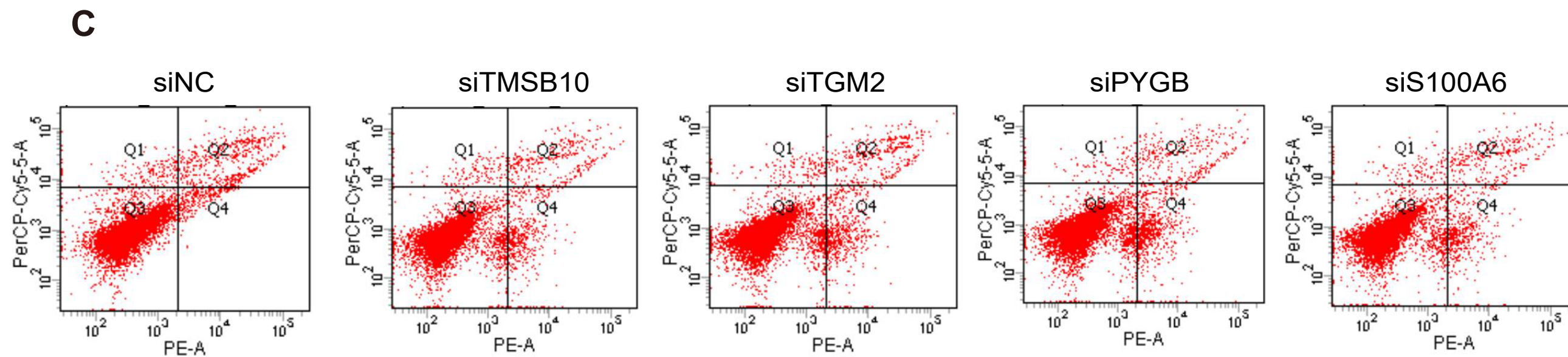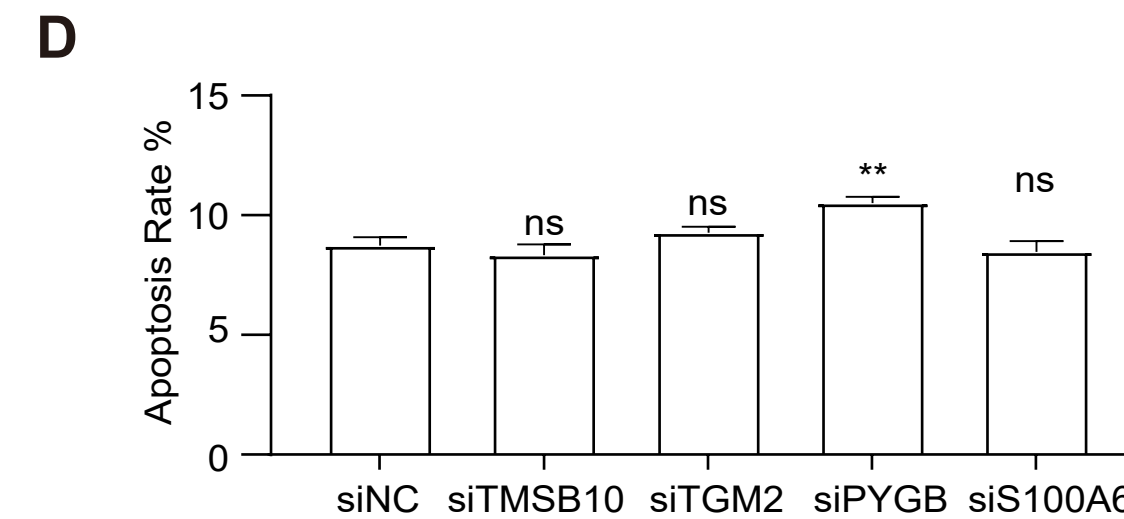

Supplement: Supplementary file 1 — Supporting Information [file PRCA-19-e202400010-s001.pdf]

A

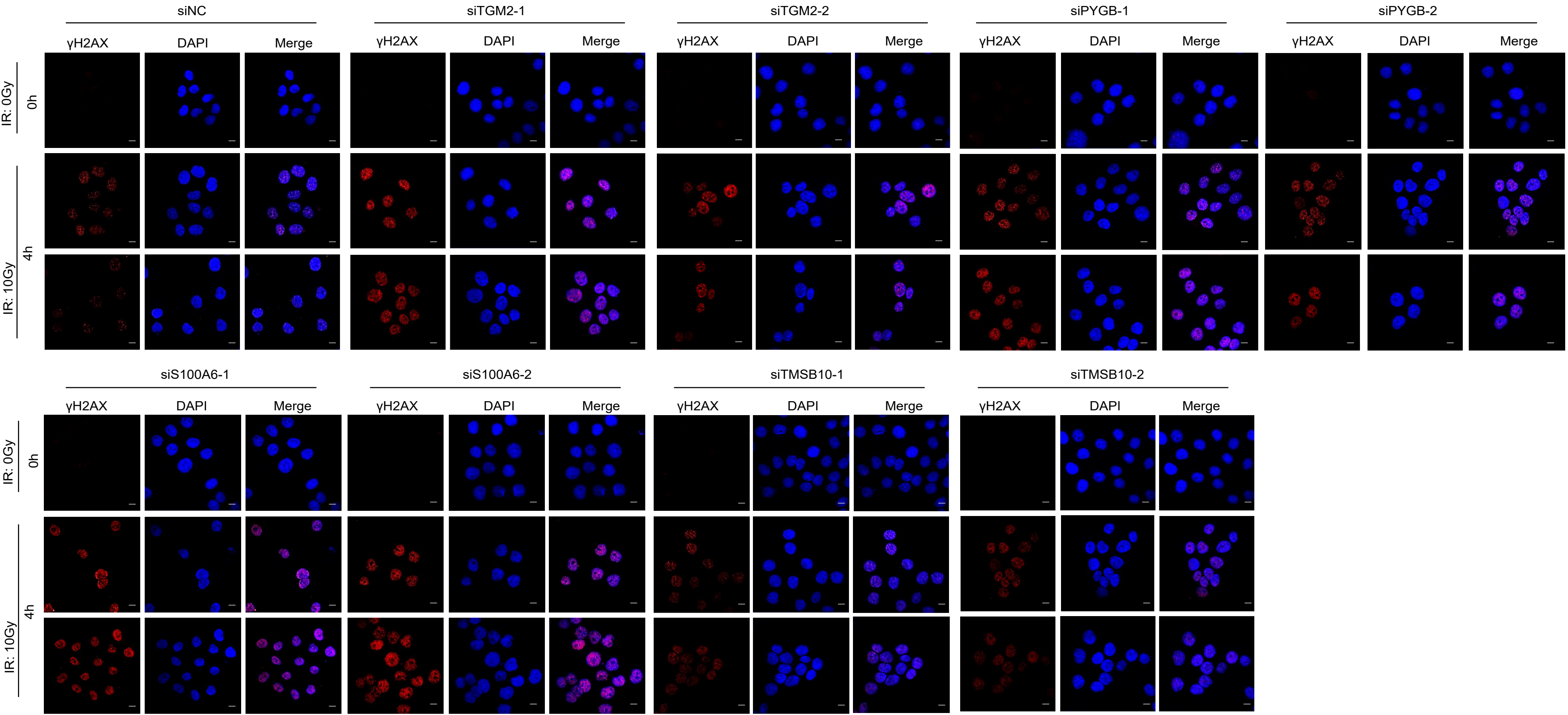

Supplement: Supplementary file 2 — Supporting Information [file PRCA-19-e202400010-s002.pdf]
